# The effects of seasons and weather on sleep patterns measured through longitudinal multimodal sensing

**DOI:** 10.1038/s41746-021-00435-2

**Published:** 2021-04-28

**Authors:** Stephen M. Mattingly, Ted Grover, Gonzalo J. Martinez, Talayeh Aledavood, Pablo Robles-Granda, Kari Nies, Aaron Striegel, Gloria Mark

**Affiliations:** 1 Department of Computer Science & Engineering, University of Notre Dame, Notre Dame, IN USA; 2 Department of Informatics, University of California, Irvine, CA USA; 3 Department of Computer Science, Aalto University, Espoo, Finland

**Keywords:** Human behaviour, Psychology and behaviour

## Abstract

Previous studies of seasonal effects on sleep have yielded unclear results, likely due to methodological differences and limitations in data size and/or quality. We measured the sleep habits of 216 individuals across the U.S. over four seasons for slightly over a year using objective, continuous, and unobtrusive measures of sleep and local weather. In addition, we controlled for demographics and trait-like constructs previously identified to correlate with sleep behavior. We investigated seasonal and weather effects of sleep duration, bedtime, and wake time. We found several small but statistically significant effects of seasonal and weather effects on sleep patterns. We observe the strongest seasonal effects for wake time and sleep duration, especially during the spring season: wake times are earlier, and sleep duration decreases (compared to the reference season winter). Sleep duration also modestly decreases when day lengths get longer (between the winter and summer solstice). Bedtimes and wake times tend to be slightly later as outdoor temperature increases.

## Introduction

Sleep is vital to health^1–4^, mood^5–9^, cognitive performance^10–12^, work quality^13–16^, and social life^17–20^. Given the importance of sleep, many contributing factors to sleep have been identified, including (but not limited to) mood^21,22^, personality^23,24^, sleep quality^25,26^, chronotype^18,27–29^, and demographic information such as age, sex, income^30–35^, and homeostatic sleep need (for a review, see refs. ^36,37^).

Sleep behavior is influenced by circadian processes, i.e., in a 24-h cycle^38–44^. Circadian systems rely on internal timekeeping cells^44^. These “clock cells” synchronize with the environment via “zeitgebers”^45^ or “time clues” such as light and ambient temperature and serve as a circadian pacemaker to coordinate other circadian responses to optimize to the environment^46–53^, e.g., in humans to promote sleep when it is dark and to be awake when it is light, to be asleep at the lowest point of core body temperature^43,54–56^, and/or to avoid extremes in ambient temperature that can impair sleep^57–61^. The circadian pacemaker changes body temperature^43,60,62,63^ and melatonin^64–66^, a hormone that promotes and maintains sleep, to assist in sleep regulation and wake cycles. In animal models, this pacemaker can adjust seasonally, in general increasing wake duration during longer periods of light and warmer temperatures (e.g., summer) and decreasing wake duration during periods with less light and lower temperatures (e.g., winter)^67–72^. In humans, it is clear that artificial light interacts with circadian systems^73–76^ (for a review, see Duffy and Wright^52^), but it is unclear if artificial light suppresses^77^ or interacts^78^ with seasonal variations in circadian mechanisms, and further, if these effects result in observed seasonal variations in sleep parameters such as sleep duration^79^.

Thus, in real-world environments with artificial light, it is not clear whether seasonal variations would affect sleep. In fact, studies examining seasonal effects on sleep have produced mixed findings. While few laboratory studies explicitly examine seasonality in humans, Wehr et al.^77^ asked 21 males (between ages 20 and 50) to record light exposure and sleep three days prior to an experimental study that gathered body temperature and hormone samples in the winter and the summer. Light sensors detected seasonal differences, but no effect of season was found on sleep duration or melatonin secretion. In a similar study, Honma et al.^79^ studied ten males (between ages 20 and 28) in an experimental living facility for 4 days each season with environmental control, sleep measurement, and exposure to natural light. They report no effect of season on sleep duration, but report earlier sleep onset and offset in winter compared to summer.

Studies using wearable sensors use objective measures but generally small sample sizes over brief periods and also have demonstrated ambiguous seasonal effects. For instance, O’Connell et al.^80^ used wearables and sleep diaries to track sleep for 1 week each season with 46 adults, and report no significant seasonal effects, while Lehnkering and Siegmund^81^ found that participants slept longer in autumn than in spring, with no seasonal effects on the bed or wake time with 34 participants for 15 days in spring and autumn. In preindustrial societies, De la Iglesia et al.^74^ examined 44 participants in hunter-gatherer tribes with and without access to electricity for one week in summer and/or winter. They report longer sleep durations in winter for both groups, but no differences in bedtime between seasons. When collapsing across both groups, they report a later wake time in summer. With 72 participants in three preindustrial societies examined between 8 and 28 days in summer and winter, sleep duration was found to be longer in winter, with earlier bedtimes and wake times in winter compared to summer (Yetish et al.^82^). Small seasonal effects on sleep duration have been reported in 8–11-year-olds measured for 7 days in three seasons^83^ and in 50–64-year-olds (but not 64–75 year-olds or 75+ year-olds)^58^, with each person measured one week but in different seasons. Participants’ data were then combined to calculate the seasonal effects.

Self-report and data repository studies (e.g., Centers for Disease Control Behavioral Risk Factor Surveillance System, http://www.cdc.gov/brfss) tend to be much larger and generally do show seasonal effects, at least for some subgroups, though they often lack bed and wake time data. For instance, Allebrandt et al.^28^ evaluated health records of 9765 participants and found that evening chronotypes had seasonal variability in sleep duration, but morning chronotypes did not. Using survey and meteorological data^57^, found that participants showed a higher rate of insufficient sleep in summer on anomalously warm nights, especially for low SES and those older than 65, but otherwise no seasonal effects. Comparing sleep in different latitudes, Friborg et al.^84^ collected sleep diaries of 330 adults for 1 week in summer and winter from Ghana (where day length increases 0.3 h from winter to summer), and Norway (which increases 11.7 h). No seasonal effects were observed for Ghanaians, while Norwegians had later bed and wake times on summer weekdays (but not weekends) with no significant difference in sleep duration. Thorleifsdottir et al.^85^ followed 668 children for 10 years who kept sleep diaries. The youngest children showed longer sleep, earlier bedtimes, and later wake times in winter compared to spring. Effects weaken with age, e.g., significant seasonal effects on sleep duration for those under 5 years old, while on weekends only for those aged 5–10, and none for older than 10 years old. This is mirrored by parental surveys about 9–12-year-old children in the US^86^, in which parents endorsed that their children slept more in winter, especially for girls. These effects were more prevalent in northern compared to southern latitudes and were reduced for older relatives to younger children. Nonwearable large-scale sensor studies also demonstrate seasonal effects. For instance, data from the “Sleep Cycle” app^87^ demonstrated teens sleep more in summer (possibly due to a break from school), and that young adults showed longer sleep durations in the winter relative to summer. Japanese users of a biomotion sensor demonstrated seasonal effects on wake time, with later wake times in winter than in summer, especially for weekends^88^. Using call records, longer sleep durations were found in winter, with more variability based on latitude^34^.

In sum, if seasonal effects are detected, sleep is found to be longer in winter and shorter in summer, likely due to increased day length and/or increased temperature, and with effects being particularly pronounced in children or the elderly^57,58,83,85,87,89^, preindustrial societies^74,82^, or in the absence of artificial light^73^, though these trends may interact with school or work demands (see refs. ^58,85,87^).

The lack of clarity in results is likely due to methodological differences and study limitations. Differences between study settings (e.g., laboratory vs. observational, industrial vs. preindustrial), the choice of datasets (wearable, self-report, and cell phone data), and the population studied (close vs. far from the equator, children vs. adults, and evening vs. morning chronotypes) may contribute to why sleep factors are found to be significantly different due to seasonal effects. Some studies only examine a small time window, such as one to 2 weeks per season^74,80,81,83^, and thus may lack the temporal resolution to detect seasonal effects, especially for fall and spring. Large-scale studies tend to rely on self-report^57,58,85^, which can be subject to memory biases or are collected from short time windows aggregated across many years^28,57,58,85,90^. The effects found in laboratory studies may not be observable in day-to-day life, may not generalize to preindustrial settings, or may only apply to specific subgroups or cultures^79,90–92^. Furthermore, seasons may have weather effects that affect sleep, e.g., snow, cloudy days, or rain (see Rifkin et al.^93^ for a meta-analysis), yet prior work on seasonal effects does not address variations in weather that are associated with seasons.

The goal of the current study is to investigate the detailed effects of seasons and weather on sleep by countering the methodological limitations of past studies. We investigated different parameters of daily sleep habits (sleep duration, bedtime, and wake time). What differentiates our study from nearly all of the past sleep studies shown in Table 1 is the use of objective, continuous, and longitudinal measures of sleep. Using wearable devices, we measured the in-situ sleep habits of 216 individuals across the U.S. across four seasons using daily monitoring of sleep and localized environmental measures, while controlling for a range of trait-like constructs previously identified to correlate with sleep behavior. Other than temperature, most weather features have been overlooked by other studies, while we were able to collect daily localized weather. This comprehensive approach allowed us to determine whether seasons and weather influence sleep in the industrial world and at the same time provides a more unifying perspective about sleep that is contextualized with respect to previous studies.

**Table 1:** Literature review of seasonal effects on sleep.

Author	Methods	Population/topic	Overall *N*/duration	Finding(s)		
				Sleep duration	Sleep onset/bedtime	Sleep offset/wake time
Allebrandt et al.^28^	Observational/survey	European adults/chronotype measurement	*N* = 9765/variable depending on sub cohort	Evening types show seasonal variability, morning types do not	NA	NA
Monsivais et al.^34^	In situ/call detail records (CDRs)	Subscribers to Telecommunication company in a Southern European country/effects on seasons and day length on sleep duration.	*N* = ~1 million/12 months	Increased in winter compared to summer, larger seasonal variability in sleep duration for southern vs northern cities	NA	NA
Stothard et al.^73^	Observational/actigraphy/light sensor/melatonin	physically active adults/circadian effects while camping	*N* = 5*, 14**/ *2 weeks in winter, **1 week in summer	2.5 h longer sleep duration during camping, both seasons	Electric environment: NA. Camping, marginally earlier melatonin offset in winter than summer	Electric environment: NA. Camping, later melatonin offset in winter than summer
de la Iglesia et al.^74^	In situ/actigraphy/sleep diaries	Neighboring hunter and gatherers; one with access to electricity/effects of electricity and season on sleep.	*N* = 44/7 days in summer or winter	Increased in winter compared to summer for both. Shorter duration for electricity access.	Sleep onset later in electricity; no effect of season	Sleep offset/rise time later in summer collapsed across community. No effect of electricity
Wehr et al.^77^	Laboratory/light sensor/melatonin/survey	Males/assessing artificial light on melatonin production	*N* = 21/48 h awake in summer and winter for 1 year. Participants were recruited across 4 years	no difference in self-reported sleep; no effect of light exposure on melatonin.	NA	NA
Adamsson et al.^78^	Observational/light sensor/melatonin	Swedish office workers/seasonal variations in melatonin and cortisol	*N* = 15/3 days per month across 1 year	Study findings: (note; no sleep effects reported) Amount and time of day of light exposure vary seasonally with access to electricity. Light exposure was highest in summer and lowest in winter. In addition, peak melatonin production was highest in winter.		
Honma et al.^79^	laboratory/PSG/self-report/physiology	20–28 yr old males in experimental living facility/ test circadian biomarkers	*N* = 10/4 days; once per season	No difference	Earlier onset in winter than summer	Earlier offset in winter than summer
Yetish et al.^82^	In situ/actigraphy	Three preindustrial societies/natural sleep without electricity.	*N* = 72/8 to 28 days, summer and winter	Increased in winter compared to summer.	Earlier in winter than summer.	Slightly earlier in winter than summer
Thorleifsdottir et al.^85^	Observational/sleep diary	children and adolesents/sleep habits	*N* = 668 children/10 years	shorter sleep in spring than in winter under age 5; similar effect age 5–10 (weekends only). Suggested due to social and school factors.	earlier bedtimes in winter than in spring under age 10 during weekdays. Ages 6–10 and 16–19 show same on weekends	later wake-up times in winter than in spring under age 10. age 16–19 show similar effects on weekends
Obradovich et al.^57^	Survey/meteorological data	USA/effect of nighttime temperature and climate change on sleep.	*N* = 765 K/9 years	Increased percentage of nights of insufficient sleep on anomalously warm nights; summer is the only significant season. effects of temp on sleep are higher in age 65+ and low socioeconomic status.	NA	NA
Cepeda et al.^58^	Observational/wearable	Middle age (50–64 yr), young elderly (65–74 yr), Old-elderly (75 + yr)	*N* = 1116/7 days with data collection spanning 5 years	31 min more of sleep in winter; middle age only. For the differences in middle age; 49% explained variance is due to temp changes. 5% due to light.	NA	NA
Friborg et al.^84^	Observational/self-report	Adults from Ghana and Norway/sunlight differences in sleep; ghana = 0.3 h between summer and winter, Norway = 11.7	*N* = 330/1 week in summer and winter	No difference	Ghana: no difference. Norway: weekday bedtime 12 min later in summer vs winter (but not weekend)	Ghana: no difference. Norway: weekday 32 min later in summer compared to winter (but not weekend)
Hjorth et al.^83^	Observational/actigraphy/sleep diary	8–11-year-old Danish children; relationship between season, sleep and physical activity.	*N* = 730/7 days in spring, winter, fall	2% (12 min) more sleep in winter relative to spring, no difference between fall and winter.	NA	NA
O Connell et al.^80^	observational/actigraphy*/ self-report**/meteorological * for activity ** for sleep	adults in UK/monitor physical activity, sedentary behavior, and sleep in all four seasons	*N* = 46/7 days sleep diary for 4 seasons	No significant differences	NA	NA
Lehnkering & Siegmund^81^	observational/actigraphy / self-report	Medical students/chronotype, season, and sex effects	*N* = 34/ 15 days in spring and autumn.	18 mins more in autumn relative to spring	No effect	No effect
Webb & Ades^91^	laboratory/PSG/meteorological	Naval aviation trainees/effects of barometric pressure on sleep	*N* = 547/9 months	NA	Significant Increase or decrease from the mean barometric pressure increased sleep onset (earlier fall asleep time), but no control for daylight length, or season.	NA
Crowley et al.^92^	Experimental/objective/ wearables	Adult employees/increase wellness	*N* = 510/1 year	Mixed: continuous monthly increase of sleep, both from fall into winter and winter into spring, likely due to experimental intervention rather than the season.	NA	NA
Robbins et al.^87^	Observational/“Sleep Cycle” app: sleep duration, quality, location, demographics	Sleep Cycle app users in New York City, aged 14–85	*N* = 160,963 (2,161,067 nights of data)/4 years.	Age 14–18 sleep the most during summer. Age 18–25 sleep more in winter and less during summer. Age 26–65 sleep more in autumn/winter than in spring/summer. Age 65–84 males slept longest in winter/autumn, females slept longest in autumn and shortest in spring.	NA	NA
Hashizaki et al.^88^	Observational/contactless biomotion sensor via radio waves	Japanese biomotion sensor users between aged 20 and 79	*N* = 1856 (691,161 nights of data)/3 years.	NA	Stable across year	Significantly varies; later in winter, earlier in summer. Especially apparent on weekends
Carskadon, M. & Acebo, C.^86^	Parental survey	Survey to parent about children age 9–12	*N* = 1680	More parents report children sleep longer in winter than in fall or spring.	NA	NA

## Results

### Participants

**Fig. 1:**
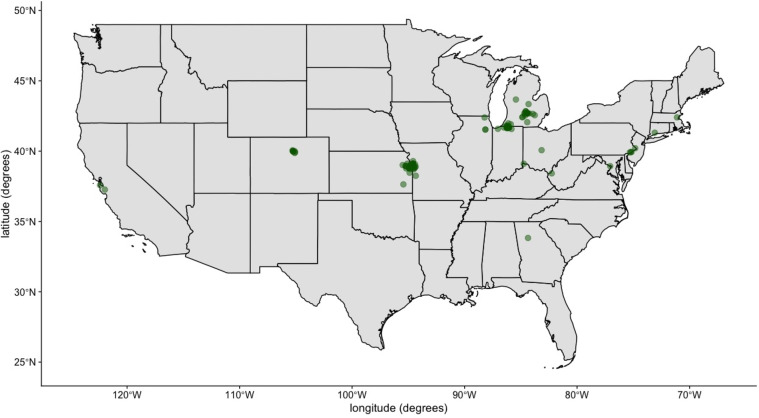
Geolocations of the participants in the study (*N* = 216). This map was obtained from the U.S. Census Bureau that permits the free use of the map for publication (https://catalog.data.gov/dataset/2013-cartographic-boundary-file-state-for-united-states-1-20000000).

**Fig. 2:**
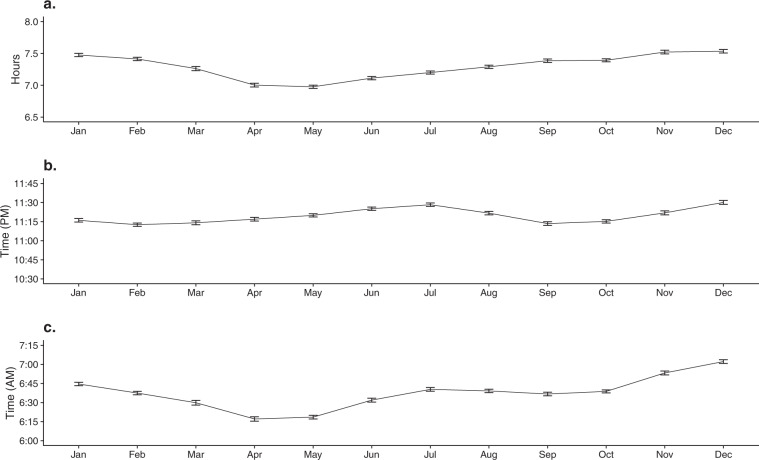
Sleep parameters per month over the study length. Each point on the graph is produced by filtering all data points that exist for that month and computing the mean of the given sleep metric. Error bars represent the standard error of the mean. **a** Average monthly adjusted sleep duration. **b** Average monthy adjusted bedtime. **c** Average monthly adjusted wake time.

After controlling for missing data (see “Methods” section), we used a final dataset of 51,836 entries of sleep data from 216 participants. Table 2 presents a full summary of demographic and psychological trait statistics for our final participant set, and Fig. 1 shows the geospatial distribution of our participants across the USA. Figure 2 presents the average sleep duration (Fig. 2a), bedtime (Fig. 2b), and wake time (Fig. 2c) over the year-long study duration. Averages in Fig. 2 were calculated from those participants who had the data for each month. Thus, the participant composition across each month may vary slightly. To ensure that the variability in the number of samples across seasons does not explain the differences between seasons, we verified that there was an approximately uniform distribution of daily observations.

**Table 2:** Demographic and psychological trait statistics for the participant pool (*N* = 216).

Categorical demographic statistics				
Gender	68.1% (147) male, 31.9% (69) female			
Organization	52.3% (113) Org O1, 11.1% (24) Org O2, 9.7% (21) Org O3, 26.9% (58) Org U			
Supervisor status	75% (162) non-supervisors, 25% (54) supervisors			

Continuous demographic and psychological trait statistics				
Measure	Min	Max	Mean	Std. dev
Age	21	63	34.9	9.10
Affect balance	−16	36	17.3	8.32
Openness	11.67	50	38.48	6.23
Conscientiousness	19.17	50	38.77	6.77
Extraversion	16.67	47.5	33.33	6.64
Agreeableness	20.83	50	38.79	5.56
Neuroticism	10	47.5	23.69	7.89
psqi	3	17	6.52	2.12
Chronotype	28	74	53.86	9.64

### Sleep duration

The sleep duration results for each of the nested models are presented in Table 3.

**Table 3:** Results for demographic and psychological traits (Model 1), seasons (Model 2), and weather variables (Model 3) predicting sleep duration.

	Model 1				Model 2				Model 3			
Predictors	Est	Std. beta	CI	Std. CI	Est	Std. beta	CI	Std. CI	Est	Std. beta	CI	std. CI
(Intercept)	5.60^*^	**0.08**	**0.82 to 10.37**	**−0.05 to 0.21**	6.34^**^	**0.11**	**1.56 to 11.12**	**−0.03 to 0.24**	6.30^**^	**0.11**	**1.52 to 11.08**	**−0.03 to 0.24**
Age	0.00	0.02	−0.01 to 0.02	−0.03 to 0.08	0.00	0.02	−0.01 to 0.02	−0.03 to 0.08	0.00	0.02	−0.01 to 0.02	−0.03 to 0.08
Gender (male)	−0.16	−0.09	−0.39 to 0.07	−0.22 to 0.04	−0.16	−0.09	−0.39 to 0.07	−0.22 to 0.04	−0.16	−0.09	−0.39 to 0.07	−0.22 to 0.04
Affect balance	−0.01	−0.03	−0.02 to 0.01	−0.11 to 0.05	−0.01	−0.03	−0.02 to 0.01	−0.11 to 0.05	−0.01	−0.03	−0.02 to 0.01	−0.11 to 0.05
Openness	0.00	0.00	−0.15 to 0.16	−0.05 to 0.06	0.00	0.00	−0.15 to 0.16	−0.05 to 0.06	0.01	0.00	−0.15 to 0.16	−0.05 to 0.06
Conscientiousness	0.03	0.01	−0.13 to 0.19	−0.05 to 0.07	0.03	0.01	−0.13 to 0.19	−0.05 to 0.07	0.03	0.01	−0.13 to 0.19	−0.05 to 0.07
Agreeableness	0.02	0.01	−0.17 to 0.20	−0.05 to 0.06	0.02	0.01	−0.17 to 0.20	−0.05 to 0.06	0.02	0.01	−0.17 to 0.20	−0.05 to 0.06
Extraversion	−0.15	−0.06	−0.31 to 0.01	−0.12 to 0.01	−0.15	−0.06	−0.31 to 0.01	−0.12 to 0.00	−0.15	−0.06	−0.31 to 0.01	−0.12 to 0.00
Neuroticism	0.05	0.02	−0.10 to 0.21	−0.05 to 0.09	0.05	0.02	−0.10 to 0.21	−0.05 to 0.09	0.05	0.02	−0.10 to 0.21	−0.05 to 0.09
Organization (O2)	−0.11	−0.07	−0.54 to 0.31	−0.31 to 0.18	−0.13	−0.07	−0.56 to 0.30	−0.32 to 0.17	−0.12	−0.07	−0.55 to 0.31	−0.31 to 0.17
Organization (O3)	−0.08	−0.05	−0.51 to 0.35	−0.29 to 0.20	−0.09	−0.05	−0.51 to 0.34	−0.29 to 0.19	−0.09	−0.05	−0.51 to 0.34	−0.29 to 0.19
Organization (U)	−0.02	−0.01	−0.26 to 0.23	−0.15 to 0.13	−0.03	−0.02	−0.28 to 0.22	−0.16 to 0.13	−0.02	−0.01	−0.27 to 0.22	−0.16 to 0.13
Supervise (yes)	0.01	0.00	−0.21 to 0.22	−0.12 to 0.13	0.01	0.00	−0.21 to 0.22	−0.12 to 0.13	0.01	0.00	−0.21 to 0.22	−0.12 to 0.13
Latitude	0.02	0.02	−0.08 to 0.12	−0.06 to 0.10	0.02	0.02	−0.07 to 0.12	−0.06 to 0.10	0.02	0.02	−0.07 to 0.12	−0.06 to 0.10
Longitude	−0.01	−0.02	−0.03 to 0.01	−0.09 to 0.05	−0.01	−0.02	−0.03 to 0.01	−0.09 to 0.05	−0.01	−0.02	−0.03 to 0.01	−0.09 to 0.04
psqi	0.00	0.00	−0.05 to 0.05	−0.06 to 0.06	0.00	0.00	−0.05 to 0.05	−0.06 to 0.06	0.00	0.00	−0.05 to 0.05	−0.06 to 0.06
Chronotype	0.01	0.05	−0.00 to 0.02	−0.00 to 0.11	0.01	0.05	−0.00 to 0.02	−0.00 to 0.11	0.01	0.05	−0.00 to 0.02	−0.00 to 0.11
Season (fall)					−0.03	−0.02	−0.07 to 0.01	−0.04 to 0.01	−0.02	−0.01	−0.07 to 0.02	−0.04 to 0.01
Season (spring)					−0.21^***^	**−0.12**	**−0.28 to −0.14**	**−0.16 to −0.08**	−0.21^***^	**−0.12**	**−0.28 to −0.14**	**−0.16 to −0.08**
Season (summer)					0.03	0.02	−0.04 to 0.10	−0.02–0.06	0.03	0.02	−0.04 to 0.10	−0.02 to 0.06
Day length					−0.07^***^	**−0.07**	**−0.08 to −0.05**	**−0.09 to −0.06**	−0.06^***^	**−0.07**	**−0.08 to −0.04**	**−0.09 to −0.05**
Outdoor temperature principal component									0.00	0.00	−0.00 to 0.00	−0.02 to 0.02
Wind principal component									0.00	0.00	−0.01 to 0.01	−0.01 to 0.01
Humidity-cloudcover principal component									0.01	0.01	−0.00 to 0.02	−0.00 to 0.02
*Random effects*												
σ^2^	2.66				2.63				2.63			
*τ*_00_	0.41_participant_				0.41_participant_				0.41_participant_			
ICC	0.13				0.14				0.14			
BIC	198,904				198,317				198374			
*N*	216_participant_				216_participant_				216_participant_			
Observations	51,836				51,836				51,836			
Marginal *R*^2^/conditional *R*^2^	0.012/0.144				0.023/0.155				0.023/0.155			

*P < 0.05, **P < 0.01, ***P < 0.001.

The reference categories for gender, organization,supervise, time zone, and season are Female, Organization O1, non-supervisors,central time zone, and winter season respectively. Est gives the unstandardized beta coefficient, std. beta gives the standardized beta coefficient, CI gives the unstandardized confidence interval, while std. CI gives the standardized confidence interval. Significant coefficients are highlighted in bold.

In the full model including demographic, psychological traits, seasonal and weather variables (Model 3), only day length and spring season were statistically significantly associated with sleep duration (Table 3), with a fixed-effect variance explained (pseudo-*R*^2^) of 0.02 and conditional variance explained (pseudo-*R*^2^) of 0.16. The intraclass correlation coefficient (ICC) for the full model was 0.14, indicating that the random effects of participants explained ~14% of the total variance explained by the model. The unstandardized beta coefficients of this model (Table 3) show that sleep duration decreases with increases in day length, and every extra hour of day length associated with a decrease in sleep duration of 3.6 min (95% C.I. (−2.4, −4.8), *P* < .001). In addition, spring was associated with a sleep duration that decreased by 12.6 min (95% C.I. (−16.8, −8.4), *P* < .001) (compared to the reference season winter). In comparing all three models, the results suggest that demographic, psychological traits, seasonal, and weather variables explained little of the variance in sleep duration in our data. Using one-sided *F*-tests for variance comparison between the models, we saw that the seasonal model (Model 2) explains significantly more variance in sleep duration than the model with demographics and psychological traits (*F* = 166.09, *P* < 0.001), and the addition of weather variables (temperature, wind, and humidity/cloud cover) in the full model (Model 3) did not explain significantly more variance over Model 2, which includes seasonal variables (*F* = 1.22, *P* = 0.30). The seasonal model (Model 2) had the lowest BIC value of all models tested indicating that it was the best-fitting model.

### Bedtime

Bedtime results for each of the nested models are presented in Table 4. In the full model, including demographic, psychological traits, seasonal, and weather variables (Model 3), day length, seasons (spring and summer), temperature weather component, age, openness, and chronotype are statistically significantly associated with bedtime (Table 4), with a fixed-effect variance explained (pseudo-*R*^2^) of 0.12 and conditional variance explained (pseudo-*R*^2^) of 0.30. The ICC for the full model was 0.22, indicating that the random effects of participants explained ~22% of the total variance explained by the model. From the unstandardized beta coefficients (Table 4), in the full model, we see that every extra hour of day length results in bedtimes that are 1.8 min earlier (95% C.I. (−2.4, −0.6), *P* < 0.001). Despite this negative effect of day length, there is an offsetting effect of season, such that spring season delays bedtimes by 4.2 min (95% C.I. (0.6, 7.2), *P* = 0.018), and summer season delays bedtimes by 6 min (95% C.I. (2.4, 9), *P* = 0.001) (compared to the winter season). A unit increase in the temperature principal component corresponds to a bedtime 0.6 min later (95% C.I. (0, 1.2), *P* < .001). For age effects, each year older is associated with bedtime 0.6 min earlier (95% C.I. (−1.2, 0), *P* = 0.041), each point higher in Openness, as scored by the BFI-10, is associated with bedtime 11.4 min later (95% C.I. (1.8, 20.4), *P* = 0.022), and each point higher in chronotype, as assessed by the MEQ, is associated with a bedtime that is 3 min earlier (95% C.I. (−3.6, −2.4), *P* < 0.001). Comparing all three models, the results suggest that demographic traits, stress, seasonal, and weather variables explain modest amounts of variance in bedtime in our data. Using one-sided *F*-tests for comparing variance between the models shows that the seasonal model (Model 2) explains significantly more variance in bedtime than the model with demographics and psychological traits (*F* = 12.04, *P* < 0.001). The addition of weather variables included in the full model (Model 3) explains only minimally more variance over the seasonal model (Model 2) (*F* = 4.66, *P* = 0.003). However, the demographics and psychological traits model (Model 1) had the lowest BIC value of all models tested, indicating it is the best-fitting model.

**Table 4:** Results for demographic and psychological traits (Model 1), seasonal (Model 2), and weather variables (Model 3) predicting bedtime.

	Model 1				Model 2				Model 3			
Predictors	Est	Std. beta	CI	Std. CI	Est	Std. beta	CI	Std. CI	Est	Std. beta	CI	Std. CI
(Intercept)	3.48	−0.13	−1.50 to 8.46	−0.29 to 0.03	3.55	−0.17	−1.43 to 8.54	−0.32 to −0.01	3.61	−0.15	−1.38 to 8.59	−0.31 to 0.00
Age	−0.01^*^	**−0.07**	**−0.02 to −0.00**	**−0.13 to −0.00**	−0.01^*^	**−0.07**	**−0.02 to −0.00**	**−0.13 to −0.00**	−0.01^*^	**−0.07**	**−0.02 to −0.00**	**−0.13 to −0.00**
Gender (male)	0.16	0.10	−0.09 to 0.40	−0.06 to 0.26	0.16	0.10	−0.09 to 0.40	−0.06 to 0.26	0.16	0.10	−0.09 to 0.40	−0.06 to 0.26
Affect balance	−0.00	−0.01	−0.02 to 0.02	−0.10 to 0.09	−0.00	−0.01	−0.02 to 0.02	−0.10 to 0.09	−0.00	−0.01	−0.02 to 0.02	−0.10 to 0.09
Openness	0.19^*^	**0.08**	**0.03 to 0.34**	**0.01 to 0.14**	0.19^*^	**0.08**	**0.03 to 0.34**	**0.01 to 0.14**	0.19^*^	**0.08**	**0.03 to 0.34**	**0.01 to 0.14**
Conscientiousness	−0.07	−0.03	−0.24 to 0.09	−0.11 to 0.04	−0.07	−0.03	−0.24 to 0.09	−0.11 to 0.04	−0.07	−0.03	−0.24 to 0.09	−0.11 to 0.04
Agreeableness	−0.08	−0.03	−0.27 to 0.12	−0.10–0.04	−0.08	−0.03	−0.27 to 0.12	−0.10 to 0.04	−0.07	−0.03	−0.27 to 0.12	−0.10 to 0.04
Extraversion	0.09	0.04	−0.08 to 0.26	−0.03 to 0.11	0.09	0.04	−0.08 to 0.26	−0.03 to 0.11	0.09	0.04	−0.08 to 0.26	−0.03 to 0.11
Neuroticism	−0.04	−0.02	−0.20 to 0.13	−0.10 to 0.06	−0.04	−0.02	−0.20 to 0.13	−0.10 to 0.06	−0.04	−0.02	−0.20 to 0.13	−0.10 to 0.07
Organization (O2)	0.45	0.29	−0.00 to 0.89	−0.00 to 0.58	0.45	0.29	−0.00 to 0.89	−0.00 to 0.58	0.44	0.29	−0.00 to 0.89	−0.00 to 0.58
Organization (O3)	0.37	0.24	−0.08 to 0.81	−0.05 to 0.53	0.37	0.24	−0.08 to 0.81	−0.05 to 0.53	0.37	0.24	−0.08 to 0.81	−0.05 to 0.53
Organization (U)	0.18	0.12	−0.08 to 0.44	−0.05 to 0.29	0.18	0.12	−0.08 to 0.44	−0.05 to 0.29	0.18	0.11	−0.08 to 0.44	−0.06 to 0.28
Supervise (yes)	−0.15	−0.10	−0.37 to 0.08	−0.24 to 0.05	−0.15	−0.10	−0.38 to 0.08	−0.24 to 0.05	−0.15	−0.10	−0.38 to 0.08	−0.24 to 0.05
Latitude	−0.05	−0.05	−0.15 to 0.05	−0.15 to 0.05	−0.05	−0.05	−0.15 to 0.05	−0.15 to 0.05	−0.04	−0.04	−0.15 to 0.06	−0.14 to 0.06
Longitude	−0.00	−0.00	−0.02 to 0.02	−0.08 to 0.08	−0.00	−0.00	−0.02 to 0.02	−0.08 to 0.08	−0.00	−0.00	−0.02 to 0.02	−0.08 to 0.08
psqi	0.01	0.02	−0.03 to 0.06	−0.05 to 0.09	0.01	0.02	−0.03 to 0.06	−0.05 to 0.09	0.01	0.02	−0.04 to 0.06	−0.05 to 0.09
Chronotype	−0.05^***^	**−0.29**	**−0.06 to −0.04**	**−0.35 to −0.23**	−0.05^***^	**−0.29**	**−0.06 to −0.04**	**−0.36 to −0.23**	−0.05^***^	**−0.29**	**−0.06 to −0.04**	**−0.36 to −0.23**
Season (fall)					0.01	0.00	−0.02 to 0.04	−0.02 to 0.03	−0.02	−0.01	−0.06 to 0.02	−0.04 to 0.01
Season (spring)					0.08^**^	**0.05**	**0.02 to 0.14**	**0.01 to 0.09**	0.07^*^	**0.04**	**0.01 to 0.12**	**0.01 to 0.08**
Season (summer)					0.13^***^	**0.09**	**0.08 to 0.19**	**0.05 to 0.12**	0.10^**^	**0.06**	**0.04 to 0.15**	**0.02 to 0.10**
Day length					−0.01	−0.01	−0.02 to 0.00	−0.03 to 0.00	−0.03^***^	**−0.03**	**−0.04 to −0.01**	**−0.05 to −0.01**
Outdoor temperature principal component									0.01^***^	**0.03**	**0.00 to 0.01**	**0.01 to 0.04**
Wind principal component									0.00	0.00	−0.01 to 0.01	−0.01 to 0.01
Humidity-cloudcover principal component									−0.00	−0.00	−0.01 to 0.01	−0.01 to 0.01
*Random effects*												
σ^2^	1.65				1.65				1.65			
*τ*_00_	0.45_participant_				0.45_participant_				0.45_participant_			
ICC	0.22				0.22				0.22			
BIC	174,348				174,371				174420			
*N*	216_participant_				216_participant_				216_participant_			
Observations	51,836				51,836				51,836			
Marginal *R*^2^/conditional *R*^2^	0.122/0.311				0.122/0.312				0.123/0.312			

*P < 0.05, **P < 0.01, ***P < 0.001.

The reference categories for gender, organization, supervise, time zone, and season are Female, Organization O1, non-supervisors, central time zone, and winter season, respectively. Est gives the unstandardized beta coefficient, std. beta gives the standardized beta coefficient, CI gives the unstandardized confidence interval, while std. CI gives the standardized confidence interval. Significant coefficients are highlighted in bold.

### Wake time

Wake time results for each of the nested models are presented in Table 5. In the full model including demographic, psychological traits, seasonal and weather variables (Model 3), day length, seasons (fall, spring and summer), chronotype score, openness, and the temperature weather, principal components are statistically significantly associated with wake time (Table 5), with a fixed-effect variance explained (pseudo-*R*^2^) of 0.09 and conditional variance explained (pseudo-*R*^2^) of 0.20. The ICC for the full model was 0.12, indicating that the random effects of participants explained ~12% of the total variance explained by the model. From the unstandardized beta coefficients (Table 5), in the full model, we see that every extra hour of day length results in wake times that are 5.4 min earlier (95% C.I. (−6.6, −4.2), *P* < 0.001). The effect of the fall season is such that wake times are 1.8 min earlier (95% C.I. (−4.8, 0), *P* = 0.030), while for the spring season, wake times are 8.4 min earlier (95% C.I. (−12.8, −4.6), *P* < 0.001), and summer season wake times are delayed by 7.8 min (95% C.I. (3.6, 11.4), *P* < .001) (compared to the reference season winter). For each point higher in chronotype, as assessed by the MEQ, wake time was 2.4 min earlier (95% C.I. (−3, −1.8), *P* < 0.001). For each point increase in Openness^94^ as scored by the BFI-10, wake time was 11.4 min later (95% C.I. (3.6, 19.2), *P* = 0.004). For each unit increase temperature principal component, wake time was 0.6 min later (95% C.I. (0, 0.6), *P* < 0.001). In comparing all three models, the results suggest that demographic, psychological traits, seasonal, and weather variables explained a modest amount of the variance in wake times in our data. Using one-sided *F*-tests for comparing variance between the models, we see that the seasonal model (Model 2) explains significantly more variance in wake time than the model with demographics and traits (*F* = 185.81, *P* < 0.001), and the addition of weather variables (temperature, wind, and humidity/cloud cover) in the full model (Model 3) explains significantly more variance compared to the model with seasons included (Model 2) (*F* = 6.23, *P* < 0.001). Comparing all three models, we see that the seasonal model (Model 2) had the lowest BIC value of all models tested indicating that it was the best-fitting model.

**Table 5:** Results for demographic and psychological traits (Model 1), seasonal (Model 2), and weather variables (Model 3) predicting wake time.

	Model 1				Model 2				Model 3			
Predictors	Est	Std. beta	CI	Std. CI	Est	Std. beta	CI	Std. CI	Est	Std. beta	CI	Std. CI
(Intercept)	9.07^***^	**−0.04**	**5.07 to 13.08**	**−0.16 to 0.08**	9.89^***^	**−0.04**	**5.89 to 13.90**	**−0.16 to 0.08**	9.91^***^	**−0.03**	**5.90 to 13.92**	**−0.15 to 0.09**
Age	−0.01	−0.04	−0.02 to 0.00	−0.09 to 0.01	−0.01	−0.04	−0.02 to 0.00	−0.09 to 0.01	−0.01	−0.04	−0.02 to 0.00	−0.09 to 0.01
Gender (male)	−0.01	−0.00	−0.20 to 0.19	−0.13 to 0.12	−0.00	−0.00	−0.20 to 0.19	−0.12 to 0.12	−0.00	−0.00	−0.20 to 0.19	−0.12 to 0.12
Affect balance	−0.01	−0.04	−0.02 to 0.01	−0.12 to 0.03	−0.01	−0.04	−0.02 to 0.01	−0.11–0.03	−0.01	−0.04	−0.02 to 0.01	−0.11 to 0.03
Openness	0.19^**^	**0.07**	**0.06 to 0.32**	**0.02 to 0.12**	0.19^**^	**0.07**	**0.06 to 0.32**	**0.02 to 0.12**	0.19^**^	**0.07**	**0.06 to 0.32**	**0.02 to 0.12**
Conscientiousness	−0.04	−0.02	−0.18 to 0.09	−0.07 to 0.04	−0.04	−0.02	−0.18 to 0.09	−0.07 to 0.04	−0.04	−0.02	−0.18 to 0.09	−0.07 to 0.04
Agreeableness	−0.06	−0.02	−0.21 to 0.10	−0.07 to 0.03	−0.06	−0.02	−0.22 to 0.10	−0.07 to 0.03	−0.06	−0.02	−0.22 to 0.10	−0.07 to 0.03
Extraversion	−0.06	−0.02	−0.19 to 0.08	−0.08 to 0.03	−0.06	−0.02	−0.19 to 0.08	−0.08 to 0.03	−0.06	−0.02	−0.19 to 0.08	−0.08 to 0.03
Neuroticism	0.02	0.01	−0.12 to 0.15	−0.06 to 0.07	0.02	0.01	−0.11 to 0.15	−0.06 to 0.07	0.02	0.01	−0.11 to 0.15	−0.06 to 0.07
Organization (O2)	0.33	0.21	−0.03 to 0.69	−0.02 to 0.43	0.32	0.20	−0.04 to 0.68	−0.02 to 0.42	0.32	0.20	−0.04 to 0.68	−0.02 to 0.42
Organization (O3)	0.29	0.18	−0.07 to 0.64	−0.05 to 0.40	0.28	0.17	−0.08 to 0.64	−0.05 to 0.40	0.28	0.17	−0.08 to 0.64	−0.05 to 0.40
Organization (U)	0.16	0.10	−0.05 to 0.37	−0.03 to 0.23	0.15	0.09	−0.06 to 0.36	−0.04 to 0.22	0.15	0.09	−0.06 to 0.36	−0.04 to 0.22
Supervise (yes)	−0.14	−0.09	−0.32 to 0.04	−0.20 to 0.03	−0.14	−0.09	−0.32 to 0.04	−0.20 to 0.03	−0.14	−0.09	−0.32 to 0.04	−0.20 to 0.03
Latitude	−0.03	−0.02	−0.11 to 0.06	−0.10 to 0.05	−0.02	−0.02	−0.11 to 0.06	−0.10 to 0.05	−0.02	−0.02	−0.10 to 0.06	−0.10 to 0.06
Longitude	−0.01	−0.03	−0.02 to 0.01	−0.09 to 0.04	−0.01	−0.03	−0.02 to 0.01	−0.09 to 0.04	−0.01	−0.03	−0.02 to 0.01	−0.09 to 0.03
psqi	0.01	0.02	−0.02 to 0.05	−0.03 to 0.07	0.02	0.02	−0.02 to 0.05	−0.03 to 0.07	0.02	0.02	−0.02 to 0.05	−0.03 to 0.07
Chronotype	−0.04^***^	**−0.22**	**−0.05 to −0.03**	**−0.27 to −0.17**	−0.04^***^	**−0.22**	**−0.05 to −0.03**	**−0.27 to −0.17**	−0.04^***^	**−0.22**	**−0.05 to −0.03**	**−0.27 to −0.17**
Season (fall)					−0.02	−0.01	−0.06 to 0.01	−0.04 to 0.01	−0.04^*^	**−0.03**	**−0.08 to −0.00**	**−0.05 to −0.00**
Season (spring)					−0.13^***^	**−0.08**	**−0.19 to −0.07**	**−0.12 to −0.04**	−0.14^***^	**−0.09**	**−0.21 to −0.08**	**−0.13 to −0.05**
Season (summer)					0.16^***^	**0.10**	**0.10–0.22**	**0.06 to 0.14**	0.13^***^	**0.08**	**0.06 to 0.19**	**0.04 to 0.12**
Day length					−0.08^***^	**−0.09**	**−0.09 to −0.06**	**−0.11 to −0.08**	−0.09^***^	**−0.11**	**−0.11 to −0.07**	**−0.13 to −0.09**
Outdoor temperature principal component									0.01^***^	**0.03**	**0.00 to 0.01**	**0.01 to 0.05**
Wind principal component									0.00	0.01	−0.00 to 0.01	−0.00 to 0.02
Humidity-cloudcover principal component									0.00	0.01	−0.00 to 0.01	−0.00 to 0.02
*Random effects*												
σ^2^	2.11				2.09				2.08			
*τ*_00_	0.29_participant_				0.29_participant_				0.29_participant_			
ICC	0.12				0.12				0.12			
BIC	186,958				186,293				186336			
*N*	216_participant_				216_participant_				216_participant_			
Observations	51,836				51,836				51836			
Marginal *R*^2^/conditional *R*^2^	0.080/0.191				0.092/0.202				0.092/0.202			

*P < 0.05, **P < 0.01, ***P < 0.001.

The reference categories for gender, organization, supervise, time zone, and season are Female, Organization O1, non-supervisors, central time zone, and winter season respectively. Est gives the unstandardized beta coefficient, std. beta gives the standardized beta coefficient, CI gives the unstandardized confidence interval, while std. CI gives the standardized confidence interval. Significant coefficients are highlighted in bold.

## Discussion

We find modest seasonal effects on sleep duration, bedtime, and wake time while controlling for demographics, location, and traits. Our results, based on a large sample and continuous objective measures, replicate previous work, and show significant demographic and trait predictors of bedtime and wake time, such as age, personality, and chronotype^18,21–24,26–34^. For sleep duration, we found significant negative effects of spring, as reported by Hjorth et al.^83^ and Thorleifsdottir et al.^85^ within children and young adult populations, though our population was adults. In addition, we found significant negative effects of day length similar to Monsivais et al*.*^34^, de la Iglesia et al.^74^, and Yetish et al.^82^. When examining seasonal effects on bed and wake times, we found the effect of spring to be significantly associated with later bedtimes and earlier wake times, in the same direction as Thorleifsdottir et al.^85^. We also found the effect of summer to be significantly associated with later bedtimes, replicating findings by Thorleifsdottir et al.^85^, Honma et al.^79^, and Yetish et al.^82^, and later wake times similar to Honma et al.^79^ and Yetish et al.^82^. Our results suggest that differences in sleep duration might be more driven by differences in wake time, rather than bedtime, similar to Hashizaki et al.^88^.

In our dataset, spring had an average of 3.6 more hours of day length than winter. Using this day length difference with our unstandardized coefficients, we found that during spring, participants had approximately: 25 min shorter sleep duration, a 25-min earlier wake time, and a 2-min earlier bedtime relative to winter. Similarly for summer, with an average of 3.5 more hours of day length than winter, we saw approximately a 12-min shorter sleep duration, a 11-min earlier wake time, and no difference in bedtime relative to winter.

One possible mechanism responsible for the correlation between day length and sleep duration is melatonin, a sleep-promoting hormone. The production of melatonin is tied to light exposure and increased day length; more light inhibits melatonin production while less light increases melatonin production^52,64,66,95,96^. Thus, day length and melatonin should covary seasonally (e.g., as seen in Honma et al.^79^). In the industrial world, light and temperature can be artificially controlled and adjusted (especially in indoor spaces) in different seasons. Artificial light may suppress melatonin production, and melatonin may not actually vary seasonally^53,77,90,95,97^, though see ref. ^78^. In our study, we demonstrated that seasonal effects (and specifically, day length) are small but still present even in an industrialized nation. We also found a modest effect of the temperature principal component for both bed and wake time. Previous studies examining ambient temperature tend to focus on temperature extremes in the absence of examining day length per se^57,59–61^. Cepeda et al.^58^ explicitly examined seasonal effects, temperature, and day length, and found that 49% of the variance in sleep duration for those aged 50–64 was due to temperature and only 5% was due to light changes. However, the authors mention that 30% of the physical activity in this age group is related to occupation, which can increase exposure to extremes of temperature as in Runkle et al.^98^, and this effect disappears in older individuals. We note that our cohort of information workers primarily worked in offices, and not outdoors. However, while the age range in our sample is fairly large (between 21 and 63), we also did not find a significant effect of age. Our sample is comprised of working adults who are not affected by a seasonal school schedule and dramatic changes in sleep and hormonal systems during puberty, which can contribute to seasonal and age-based sleep effects^35,83,85,86^.

Our results help clarify the findings of past studies. Our study data are based on 51,836 observations from 216 individuals, from 33 days or more in each of the four seasons. While an exact comparison is difficult, our study has at least three times the observations compared to previous wearable-based work, e.g., Cepeda et al.^58^ *n* = 116, 7 days, Friborg et al.^84^, *n* = 330, 7 days, two seasons, and Hjorth et al.^83^, *n* = 730, 7 days, three seasons. Our more fine-grained approach demonstrates modest seasonal effects, whereas prior methodologies may not have been sensitive enough to detect these small differences. By using objective, continuous, and long-term sleep data collected in situ within participants across all four seasons, and while controlling for known demographic and psychological confounds, we could detect such differences. Our work can be seen as a link among different findings in prior studies that used different sample characteristics and measures. We controlled for a range of demographic and trait measures along with sleep and weather; it had not been clear from prior work what the effects of these different variables would be in a long-term study. Our relatively large sample enables generalizability of our results to an adult population of college-educated information workers while remaining consistent with some previous work^34,74,82,85^. Our temporal resolution allowed us to test day-to-day weather effects across all seasons and identify that temperature has a small but significant effect, while showing that other weather factors of humidity/cloud cover and wind did not have a significant effect. Our study suggests that future studies of sleep duration, bedtime, and wake time should consider seasonal and daily-level variables such as day length and temperature.

Given the relative importance of season and temperature on sleep compared to other traits and demographic information, future work aimed at optimizing bedtime, wake time, and sleep duration could focus on implications for domains related to health and well-being. For example, seasonal effects may impact those with year-round rigid school and work schedules (e.g., medical interns). Seasonal effects can inform the design of living environments that are totally artificially controlled, e.g., in Arctic stations, submarines, or spacecraft. Smart home designs could also benefit by making allowances for seasonal effects, e.g., by increasing and decreasing lighting and/or temperature on a seasonal basis. Seasonal effects on sleep could be detected in other measurement and usage information, such as network or cell phone data^34,87^, and be used to measure seasonal sleep effects or interventions in the absence of a fitness tracker.

Our study has several limitations. After filtering out participants with inadequate data, the remaining participants in our sample may have been biased in traits (e.g., conscientiousness) associated with better compliance with the study procedures. However, inadequate data could also have been due to technical issues^99^. Our participants were mostly college-educated information workers within the US. Therefore, we can only generalize our results to similar populations. The geographic locations of our participants within the US were spread across a relatively large range of latitudes and longitudes; however, only one participant was on the US west coast. Despite controlling for demographic and trait information of our participants, we were not able to control for exogenous factors (e.g., significant news events, stressful life events, work pressures, or dependents) that may have impacted sleep duration or quality over a period of time. Next, our sample consisted of information workers with flexible work schedules, which may allow more variability in bed and wake times than hourly workers. Another limitation is the use of weather data, rather than personally sensed environmental measurements. Our study cannot comment on how and to what extent participants were exposed to weather, daylight, temperature, and seasonal variation in these constructs. For instance, participants could adapt to cold temperatures with central heating or warmer clothing. However, other wearable sensor studies have determined that light and temperature variations experienced in situ predict bed and wake times^100^, and that these exposures vary seasonally even in controlled environments^101^. While participants may reduce seasonal variability in controlled environments, these efforts do not remove seasonal effects on sleep. Another limitation is the examination of sleep only during the normal work week. Indeed, it may be possible that weekend sleep (which is generally more variable and less subject to social demands) would be more affected by seasonality^88^. Future research should consider how seasonality affects weekends and the difference between weekday and weekend sleep.

The switching of clocks due to seasonal time changes (daylight savings/standard time) could potentially have affected our results. However, we excluded data for the week following daylight- saving time adjustments^102–105^. We also note that work schedules would also have adjusted with the time changes. As we used day length as a measure, adjusting both sunrise and sunset by an hour would result in the same day-length duration. Future studies should consider how sleep changes in response to DST are affected by seasons.

In conclusion, continuous tracking of objective sleep measures over the year shows that seasons do have a modest but significant effect on sleep, even after accounting for known demographic and psychological trait influences. This study helps to clarify differences in past investigations of seasonal effects. Our study suggests the value of using fine-grained temporal resolution in examining environmental effects such as seasons, day length, temperature, and weather on sleep.

## Methods

### Ethics

This study was approved by the University of Notre Dame’s IRB under protocol number 17-05-3870. All participants provided written informed consent prior to taking part in the study.

### Demographics and psychological traits

We control for several demographic (age, sex, organization, supervisory role, latitude, and longitude of home location) and trait measures, known to be associated with sleep behavior^18,21–25,27–34^, collected from a survey at the onset of the study. Psychological traits were measured from validated inventories: Positive and Negative Affect Schedule (PANAS-X)^106^ for affect balance (positive minus negative affect), Big Five Inventory (BFI)^94^ for personality, the Pittsburgh Sleep Quality Index (PSQI)^25^ for sleep quality, and Morning-Eveningness Questionnaire (MEQ)^107^ for chronotype (larger scores indicate more “morning types”, smaller scores more “evening types”).

### Sleep

To measure sleep, participants wore the Garmin VivoSmart 3 fitness band (24/7) for the year-long duration of the study. Participants’ daily sleep durations, bedtimes, and wake times were collected from the Garmin Health API (https://developer.garmin.com/health-api/overview/). Wearables can accurately detect sleep (see refs. ^108,109^), and we increased sleep measurement accuracy by leveraging phone usage and wearable-derived bedtimes, wake times, and sleep duration (see refs. ^110,111^).

### Weather and seasons

Daily weather data were collected from the World Weather Online developer API (https://www.worldweatheronline.com/developer/api/) using home location zip codes. Fourteen numeric weather variables were collected (e.g., sunrise/sunset times, temperature (including minimum, maximum, and average), humidity, cloud cover, temperature, wind speed, visibility, and pressure). Principal component analysis to reduce the dimensionality of the data yielded three principal components determined mainly by temperature, wind, and humidity/cloud cover accounting for 71.2% of the variance in the weather. Day length was calculated from sunrise/sunset times of participants’ local locations. Season start dates were defined via astronomical seasons as follows: spring—March 20, summer—June 21, fall—September 22, and winter—December 21 (https://www.weather.gov/media/ind/seasons.pdf).

### Travel

As travel can result in variable sleep patterns (e.g., change of time zone, schedules) and travel can expose participants to different weather conditions, we excluded participant travel days. The number of entrees of sleep data identified as travel days were 3261 (average of 14.8 days/person) and were excluded from the final dataset (see the section “Location sensing”).

### Location sensing

To determine participant location for weather and travel, we used two Gimbal Series 21 Bluetooth beacons that participants placed in the home and office locations. Beacon sightings recorded by the participants’ smartphones determined when they were home or at the office. Gimbal Bluetooth beacons operate in the 2.4-GHz band, allowing them to be sighted in most home and work environments^112^ and up to 100 m away in unobstructed environments (https://support.gimbal.com/hc/en-us/articles/218653567-Customizing-Beacon-Detection-RSSI). Home latitude and longitude are computed using the home beacon location data. Travel is computed using beacon sightings in conjunction with smartphone location data. We defined travel days as when they had both a lack of beacon sightings and a smartphone location more than a 300-mile distance from home or an average distance of more than 200 miles away during the day.

### Procedures

Data were collected from 649 individuals (of 757 who started the study) from across the U.S., who completed data collection of approximately a year, from February 5, 2018 to March 15, 2019. The majority of the participants were concentrated in three different organizations (denoted O1, O2, and O3), and some without a defined organization (denoted U). The characteristics of the participants, sensing streams, and full study details are described in Mattingly et al.^111^. We used data only from weekdays rather than weekends, as weekend sleep is not necessarily reflective of one’s normal sleep patterns^29,113,114^. We excluded data entries from days where participants were detected to be away from home (see travel calculations above). We also excluded the five weekdays after each daylight savings time (DST) change in our data period (March 11, 2018, November 4, 2018, and March 10, 2019), as DST changes have been shown to generally affect sleep patterns up to a week after the change^102–105^. To account for missing actigraphy data (e.g., dead battery, device not worn) and to ensure adequate data for each participant in each season, we excluded participants who had data for <50% of weekdays during any of the four seasons (i.e., at least 33 weekdays per season). We did this rather than impute missing data, as imputed data may introduce a bias for some participants, which may in turn bias our insights^115^. The resulting dataset of 216 participants after this data cleaning had 51,836 observations. Participants had a mean of 239.9 days of data (min: 184, max: 275). Participants had a mean of 61.2 days of data in the winter season (min: 33, max: 84), 57.8 days of data in the fall season (min: 42, max: 60), 57.1 days of data in the spring season (min: 33, max: 66), and 63.9 days of data in summer season (min: 41, max: 67).

### Analysis

Previous literature suggests that seasonal effects may occur on bedtime, wake time, or sleep duration independently (see refs. ^79,81,82^), which led us to run independent models for each variable. As our data consist of repeated observations of sleep data for each participant, we model our data using mixed linear-effect models. We include a random intercept effect on the participant identifier, to predict the daily sleep variables. To address our research questions, we use a hierarchical regression framework to investigate the cumulative variance of sleep duration, bedtime, and wake time using the following nested models: a Baseline (no fixed-effect variables); Model 1, adding demographic and psychological trait variables; Model 2, adding seasonal variables; Model 3, adding weather variables. For predicting bedtime, we used the daily variables (e.g., day length) for the day leading up to a given participant’s bedtime. For sleep duration and wake time, we use daily variables from the previous day (i.e., day length from the previous day predicting wake time the following morning). The general equations for the models tested are defined as follows, where *y* refers to the sleep variable of interest, y*/participant* refers to the random intercept effect on the participant identifier, $f\left( {x_1, \ldots ,x_n} \right) = c_0 + c_1x_1 + \ldots + c_nx_n$.

$$\begin{array}{l}{\mathrm{Baseline}}: = y\left| {{\mathrm{participant}} = f\left( 1 \right)} \right.\\ {\mathrm{Model}}\,{\mathrm{1:}} = y\left| {{\mathrm{participant}} = f\left( {x_1, \ldots ,x_{14}} \right)} \right.\\ {\mathrm{Model}}\,{\mathrm{2:}} = y\left| {{\mathrm{participant}} = f\left( {x_1, \ldots ,x_{14},x_{15},x_{16}} \right)} \right.\\ {\mathrm{Model}}\,{\mathrm{3:}} = y\left| {{\mathrm{participant}} = f\left( {x_1, \ldots ,x_{14},x_{15},x_{16},x_{17},x_{18},x_{19}} \right)} \right.\end{array}$$

where the independent variables are

$$\begin{array}{l} x_1: = {\mathrm{age}},\,x_2: = {\mathrm{gender}},x_3: = {\mathrm{affect}}\,{\mathrm{balance}},\,x_4: = {\mathrm{openness}},\\ x_5: = {\mathrm{conscientiousness}}, \\ {x_6: = {\mathrm{agreeableness}},x_7: = {\mathrm{extraversion}},x_8: = {\mathrm{neuroticism}},x_9: = {\mathrm{organization}},}\\ {x_{10}: = {\mathrm{supervise}},x_{11}: = {\mathrm{latitude}},x_{12}: = {\mathrm{longitude}},x_{13}: = {\mathrm{psqi}},x_{14}: = {\mathrm{MEQScore}}} \\ {x_{15}: = {\mathrm{season}},x_{16}: = {\mathrm{day}}\;{\mathrm{length}}} \hfill \\ \begin{array}{l}x_{17}: = {\mathrm{temperature}}\,{\mathrm{principal}}\,{\mathrm{component}},\,x_{18}: = {\mathrm{wind}}\,{\mathrm{principal}}\,{\mathrm{component}},\\ x_{19}: = {\mathrm{humidity}} - {\mathrm{cloudcover}}\,{\mathrm{principal}}\,{\mathrm{component}}\end{array} \end{array}$$

For each model, beta coefficients are standardized via the Gelman method whereby the estimates are reduced by dividing them by two standard deviations^116^, in order to allow direct comparison of the strengths of the effects of the variables in the model. All *P* values and 95% confidence intervals for beta coefficients are calculated using a parametric bootstrap method in order to ensure model robustness^117^. For all models, a variance inflation factor (VIF) analysis showed the max GVIF^1/(2*Df)^ for any variable to be 2.80 (day length), indicating acceptable levels of multicollinearity^118,119^. Pseudo *R*^2^ values for both marginal (fixed effects alone) and conditional (random and fixed) effects are computed using the method described by Nakagawa and Schielzeth^120^. Finally, for each dependent variable (sleep duration, bedtime, and wake time), we also use a model comparison test using Bayesian Information Criterion (BIC). We use BIC to determine the most optimal model accounting for the models’ degrees of freedom. The R programming language and packages dplyr^121^, tidyr^122^, lubridate^123^, lme4^124^, car^125^, jtools^126^, ggplot2^127^, cowplot^128^, sf^129^, and sp^130^ were used for analyses and visualizations.

### Reporting summary

Further information on research design is available in the Nature Research Reporting Summary linked to this article.

## Supplementary information

Reporting Summary

## Data Availability

The data that support the findings of this study are available from the corresponding author upon reasonable request.

## Code availability

The code that supports the findings of this study is available from the corresponding author upon reasonable request.
