# Supplementary material for: The effects of seasons and weather on sleep patterns measured through longitudinal multimodal sensing
Source: NPJ Digit Med. 2021 Apr 28;4:76. doi: 10.1038/s41746-021-00435-2 (PMC8080821; doi:10.1038/s41746-021-00435-2)
Supplement: Supplementary file 1 — Reporting Summary [file 41746_2021_435_MOESM1_ESM.pdf]

nature research

Corresponding author(s): Stephen Mattingly
Last updated by author(s): Oct 19, 2020

Reporting Summary

Nature Research wishes to improve the reproducibility of the work that we publish. This form provides structure for consistency and transparency in reporting. For further information on Nature Research policies, see our Editorial Policies and the Editorial Policy Checklist.

Statistics

For all statistical analyses, confirm that the following items are present in the figure legend, table legend, main text, or Methods section.

n/a	Confirmed	
☐	☒	The exact sample size (*n*) for each experimental group/condition, given as a discrete number and unit of measurement
☐	☒	A statement on whether measurements were taken from distinct samples or whether the same sample was measured repeatedly
☐	☒	The statistical test(s) used AND whether they are one- or two-sided<br>*Only common tests should be described solely by name; describe more complex techniques in the Methods section.*
☐	☒	A description of all covariates tested
☒	☐	A description of any assumptions or corrections, such as tests of normality and adjustment for multiple comparisons
☐	☒	A full description of the statistical parameters including central tendency (e.g. means) or other basic estimates (e.g. regression coefficient) AND variation (e.g. standard deviation) or associated estimates of uncertainty (e.g. confidence intervals)
☐	☒	For null hypothesis testing, the test statistic (e.g. *F*, *t*, *r*) with confidence intervals, effect sizes, degrees of freedom and *P* value noted<br>*Give P values as exact values whenever suitable.*
☒	☐	For Bayesian analysis, information on the choice of priors and Markov chain Monte Carlo settings
☐	☒	For hierarchical and complex designs, identification of the appropriate level for tests and full reporting of outcomes
☒	☐	Estimates of effect sizes (e.g. Cohen's *d*, Pearson's *r*), indicating how they were calculated

Our web collection on statistics for biologists contains articles on many of the points above.

Software and code

Policy information about availability of computer code

Data collection	Qualtrics for survey administration. Red Hat Enterprise Linux was used to run data storage servers. Python 2 and 3, with Pandas, Flask, Scikit-Learn, NumPy, Psycopg2, paho-mqtt. MongoDB, PostgreSQL were used to collect, organize, pre-process data. Garmin Companion SDK, Garmin Health API, and Gimbal SDK were used to collect wearable and beacon data. Android and iOS were used to collect phone and wearable data.
Data analysis	The open source "R" software package was used. packages include: dplyr, tidyr, lubridate, lme4, car, jtools, ggplot2, and cowplot were used for analyses.

For manuscripts utilizing custom algorithms or software that are central to the research but not yet described in published literature, software must be made available to editors and reviewers. We strongly encourage code deposition in a community repository (e.g. GitHub). See the Nature Research guidelines for submitting code & software for further information.

Data

Policy information about availability of data

All manuscripts must include a data availability statement. This statement should provide the following information, where applicable:

- Accession codes, unique identifiers, or web links for publicly available datasets
- A list of figures that have associated raw data
- A description of any restrictions on data availability

The data that support the findings of this study are available from the corresponding author upon reasonable request.

Field-specific reporting

Please select the one below that is the best fit for your research. If you are not sure, read the appropriate sections before making your selection.

☒ Life sciences ☐ Behavioural & social sciences ☐ Ecological, evolutionary & environmental sciences

For a reference copy of the document with all sections, see nature.com/documents/nr-reporting-summary-flat.pdf

Life sciences study design

All studies must disclose on these points even when the disclosure is negative.

Sample size	Sample sizes were determined as part of a larger work. 757 participants were enrolled, 649 completed the study. 216 had sufficient data coverage within each season.
Data exclusions	For the data used to produce the models reported in our work, the final set of participant data was reduced from 649 to 216 participants who had complete (i.e. no missing) data for both the dependent variables and covariates used in our models. Exact data exclusion criteria and processes is provided in the 'Procedures' subsection in the Methods section of our paper.
Replication	This is an observational dataset. Given the difficulty in collecting the data, no replication was attempted. However, our findings in general replicate previous studies.
Randomization	This study was observational in nature, and thus no experimental condition applies and participants were not randomly assigned to any group.
Blinding	As part of a larger work, a subset of 150 were blinded. for this work, all participants, both blinded and non-blinded were included.

Reporting for specific materials, systems and methods

We require information from authors about some types of materials, experimental systems and methods used in many studies. Here, indicate whether each material, system or method listed is relevant to your study. If you are not sure if a list item applies to your research, read the appropriate section before selecting a response.

Materials & experimental systems

n/a	Involved in the study	
☒	☐	Antibodies
☒	☐	Eukaryotic cell lines
☒	☐	Palaeontology and archaeology
☒	☐	Animals and other organisms
☐	☒	Human research participants
☒	☐	Clinical data
☒	☐	Dual use research of concern

Methods

n/a	Involved in the study	
☒	☐	ChIP-seq
☒	☐	Flow cytometry
☒	☐	MRI-based neuroimaging

Human research participants

Policy information about studies involving human research participants

Population characteristics	Please see table 2.
Recruitment	Participants were information workers required to be full time employees at the time of enrollment. Participants were recruited from primarily 3 institutions, primarily through email. This population's work demands may allow flexibility that does not occur in the broader population.
Ethics oversight	The IRB at the university of Notre Dame , protocol 17-05-3870

Note that full information on the approval of the study protocol must also be provided in the manuscript.
